# Current uses of electro-cautery lumen apposing metal stents in endoscopic ultrasound guided interventions

**DOI:** 10.3389/fmed.2022.1002031

**Published:** 2022-11-30

**Authors:** Hang Yi, Qin Liu, Song He, Li Zhong, Su-hua Wu, Xiao-dong Guo, Bo Ning

**Affiliations:** ^1^Department of Gastroenterology, The Second Affiliated Hospital of Chongqing Medical University, Chongqing, China; ^2^Department of Gastroenterology, Chongqing General Hospital, Chongqing, China

**Keywords:** electro-cautery lumen apposing metal stents, interventional endoscopic ultrasound, pancreatic fluid collection, endoscopic ultrasound-guided biliary drainage, endoscopic ultrasound-guided gastroenterostomy

## Abstract

The electro-cautery lumen apposing metal stent (EC-LAMS) is a newly developed device that integrates the electro-cautery cyctotome with the one-step metal stent delivery and releasing system in recent years. LAMS was first designed to complete the drainage of pancreatic fluid collection under endoscopic ultrasound guidance, and the technological innovation of EC-LAMS has made more off-labeled indications of endoscopic intervention for gastrointestinal diseases realized, such as abdominal fluid drainage, bile duct, or gallbladder drainage through stomach or duodenum, gastrointestinal anastomosis, and the establishment of fistulous channel for further endoscopic operation when necessary. The unique feature of this metal stent is that it has the design of a saddle shape and a large lumen, and can almost connect the adjacent structures to minimize the risk of perforation and leakage. Compared with traditional LAMS, EC-LAMS, an advanced integrated device, can greatly simplify the endoscopic process, shorten the procedure time and reduce the technical difficulty, thus it can help endoscopists complete more complex endoscopic interventions. In this review, we discuss the state of art with regard to EC-LAMS and its endoscopic process, current indications, outcomes, adverse events, and future application prospects.

## Introduction

The lumen-apposing metal stent (LAMS) is a saddle shaped metal stent with a large channel, which was first reported by Binmoeller and Shah for transluminal drainage in 2011 (1, 2). It is mainly designed for the drainage of peripancreatic fluid collections (PFCs) and has been applied in recent years. The LAMS contains high patency and provides sufficient fluid drainage, but has the limitation of relatively complicated operational steps of procedures and the use of accessories such as guide wire, catheter, cystotome, or dilation balloons under the guidance of X-ray and endoscopic ultrasound (EUS). Although the indications of LAMSs are gradually widespread, its complicated operation process and high operation difficulty limit its clinical practice. In recent years, with the progress of technological innovation, different types novel electrocautery LAMS have been developed. The unique design integrates the electro-cautery cyctotome and the metal stent releasing system, which greatly facilitates the operation steps, reduces procedural difficulties, and widely expands the clinical indications.

## Design of EC-LAMS and procedure process

Prior to the introduction of EC-LAMS, several types of conventional non-cautery-based LAMS were widely used (3, 4). The transluminal placement of a cold LAMS requires multiple over-the-wire device exchanges which may result in difficulties for endoscopists to master this technique. During the procedure of releasing a cold LAMS, a 19-G fine-needle is firstly used to enter the target lumen, and the anatomical structure of the lumen is subsequently confirmed for placing the guide wire into the cavity through contrast injection. Then the needle is exchanged for a dilation balloon (or bougie) to expand the transluminal tract to insert a stent delivery catheter and finally a LAMS is placed. Each step of this technique has potential complications. Guidewire access may be lost during instrument exchange. Removing the instrument from the wire can probably leave a step-off between the wire and the tract, which may cause leakage. Inserting an instrument along the guidewire can cause perforation and/or separation of the target and intestinal lumen. Dilation of the transluminal tract may lead to perforation and bleeding (5). For this reason, a novel stent delivery system with simple manipulation and refined procedure steps is needed and the EC-LAMS is consequently developed.

There are two types of EC-LAMS that are currently popular in clinical use: HOT AXIOS stent (5) (Boston Scientific, Marlborough, Mass, US) and HOT SPAXUS stent (6, 7) (Taewoong Medical, Gyeonggi-do, South Korea), and the parameters of them are listed in Table 1.

**Table 1:** Current electro-cautery lumen apposing metal stent on market.

**Types of stents**	**Stent length (mm)**	**Lumen diameter (mm)**	**Flange diameter (mm)**	**Delivery catheter (Fr)**
HOT AXIOS stent (Boston Scientific, Marlborough, Mass, US) (5)	8, 10, 15	6, 8, 10, 15, 20	14, 17, 21, 24, 29	9, 10.8
HOT SPAXUS stent (Taewoong Medical, Gyeonggi-do, South Korea) (6, 7)	20	8, 10, 16	23, 25, 31	10

### HOT AXIOS stent

**Figure 1:**
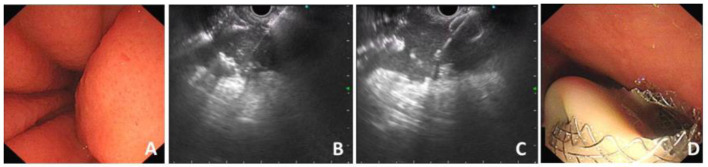
Procedure process of EC-LAMS for drainage of PFC. **(A)** Endoscopic view of compression of the posterior wall of the stomach. **(B)** Puncture of PFC with the electrocautery system under the guidance of EUS. **(C)** Release of the distal flange. **(D)** A large amount of necrotic fluid flows out through the deployed EC-LAMS.

The HOT AXIOS stent was developed to enable the endoscopist to an immediate release of the stent following an access to the target lumen with a stent-loaded delivery catheter using the electro-cautery tip under endoscopic ultrasound instead of a needle or guidewire insertion or preliminary dilation (3, 5, 8). The operation process of HOT AXIOS stent mainly includes two steps: cyst puncture and stent release. It integrates the cystotome and the stent delivery device together, without the assistance of guide wire or fluoroscopy, and is easy and fast to operate. First, the location of the target lesion to be punctured (such as pancreatic pseudocyst) is identified under EUS and the appropriate depth of cystic lesion is measured to evaluate the puncture length of the catheter. Second, directly puncture into the lumen of lesion under the guidance of EUS through the electro-cautery stent delivery catheter. Third, release the first flange and gently pull it back to make the first flange closely against the cystic wall. Finally, the proximal flange is gradually deployed within the gastrointestinal lumen with the maintain of a certain degree of traction force, so that the metal stent could expand slowly and the drainage channel is established (9) (Figure 1).

### HOT SPAXUS stent

The HOT SPAXUS stent is another EC-LAMS in popular use (6, 7). When using this stent, the target lesion is punctured using a 19-G FNA needle followed by an advancement of a 0.025/0.035-inch guidewire into the lumen. After placement of the guidewire, the transluminal tract is dilated by applying electrocautery. The stent delivery system is then advanced over the guidewire and the two flanges are immediately deployed one after another under the guidance of X-ray or endoscopic ultrasound between the lesion cyst and the gastrointestinal tract.

## Challenges of the deployment of EC-LAMS

The stent release process is critical, and improper operation may cause stent migration. Staudenmann et al. (10) reported a case of EC-LAMS translocation and dislodgement into the gastric cavity. The stent was retrieved with a biopsy forceps and then placed again by reloading the proximal end of the LAMS into the therapeutic endoscope channel and pushing the biopsy forceps to grasp the distal end of the stent to reintroduce it into the lesion. It is suggested that we should pay much attention to the deployment of the distal flange of EC-LAMS, especially not drag it too hard during the delivery procedure. The proximal flange should be released in the therapeutic channel of endoscope, and then gradually pushed into the gastrointestinal cavity under the direct observation of endoscope view so as to prevent internal leakage caused by early release of the flange.

## Indications and outcomes of EC-LAMS

### Pancreatic fluid collection (PFC) and wall-off necrosis (WON)

EC-LAMS have become the optimal choice for treatment of PFC or WON primarily related to ease of use and perceived advantage of a large lumen to facilitate drainage and direct endoscopic necrosectomy (9, 11, 12). In a nationwide survey from Italy, 97.2% of endoscopists perform LAMS positioning for PFC (13). The performance of EC-LAMS can reach high technical rate of 97.1%, clinical success rate of 88.8%, and cumulative adverse effects (AE) of 18.3% (7.4% for stent migration, 7.9% for stent occlusion and infection, 2% for major bleeding, and 1% for buried stents) (8). Factors related to higher risks of AEs include pre-procedural evidence of pancreatic duct leak/disruption, vessel alteration, requiring percutaneous drainage, or a multigate technique, and as well hospital volume is significantly associated with improved outcomes (14, 15). When comparing LAMS with plastic stent (PS) for WON drainage, LAMS was more efficacious, with a success rate of 92 vs. 84% for PS, the procedure duration was significantly shorter than PS and rates of unplanned endoscopy and surgery were both lower with LAMS approach that was, however, more costly (20,029 US dallars for LAMS vs. 15,941 US dallars for PS) (16). However, in some cohort study, LAMS was considered to be associated with significantly higher rates of procedure related bleeding and greater need for repeat endoscopic intervention, thus some experts still recommended PS drainage (17).

A recent multicenter study demonstrated that deployment of double-pigtail PSs across EC-LAMS at the time of initial drainage did not have a significant effect on clinical outcomes, adverse events, or need for reinterventions (1-pigtail vs. 2-pigtails, 7 French vs. 10 French pigtail), suggesting application of EC-LAMS alone was enough for PFC drainage (Table 2) (18).

**Table 2:** Summary of the unique characteristics of EC-LAMS compared with double pigtail plastic stents.

The large diameter of EC-LAMS facilitates better drainage of fluids or viscous contents from a cavity or organ.
By the virtue of the “apposing” characteristics, the EC-LAMS minimizes the risk of leakage.
The large lumen of EC-LAMS acts as a working channel to undertake endoscopic interventions in adjacent structures of the gastrointestinal tract.
The integrated single-step delivery system for EC-LAMS simplifies technical steps of the endoscopic procedure.

The recommendation time of removal of LAMS is 4 weeks in consensus because of increased possibilities of delayed bleeding and buried stent syndrome, but two recent multicenter studies showed conflicting results in this regard. In an Italian nationwide study from 30 centers, subgroup analysis highlighted no significant differences in terms of AEs according to the LAMS removing time (early < 4 weeks and late >4 weeks), and an 18-unit experience from UK and Ireland showed no increased rate of delayed events when the LAMS were removed beyond 4 weeks (7 weeks in average) (19, 20).

### Malignant biliary strictures when ERCP failed

Currently, EC-LAMSs with diameters of 6, 8, and 10 mm are available to simplify the placement in patients with distal malignant biliary strictures (Table 1). EUS-guided choledochoduodenostomy (EUS-CD) with EC-LAMS is usually carried out when ERCP is not possible or failed due to tumor invasion of the papilla or an inaccessible papilla caused by duodenal stenosis or prior duodenal stent placement and unsuccessful biliary cannulation (21, 22). According to the recent multiple-center data from 6 US centers, 7 French centers and 8 UK and Ireland centers, technical success rates ranged from 90.8 to 97.8%, and clinical success rates were ~93.4–100% with AE rates of 1.6–17.5% (22–24). Duodenal invasion seems to increase the risk of developing EUS-CD dysfunction, potentially representing a relative contraindication for this technique (25). Inserting an axis-orienting stent through the lumen of the LAMS may reduce the need for biliary re-interventions (23).

### Cholecystitis with high risk of surgery

EUS-guided gallbladder drainage (EUS-GBD) has been demonstrated to have similar technical and clinical success with percutaneous transhepatic gallbladder drainage (PT-GBD) for the treatment of cholecystitis in patients with high risks of surgery (2, 26, 27). Patients who undergo EUS-GBD seem to have shorter hospital stays, lower pain scores, and fewer repeated interventions, with a trend toward fewer AEs (26). Dollhopf et al. summarized 75 high-risk surgical patients who underwent EUS-GBD by EC-LAMS, the rates of technical and clinical success were 98.7 and 95.9%, respectively (28). Adverse events were encountered in 10.7% of patients of which 1.3% were intraprocedural and 9.4% were observed at follow up. Three patients without resolution of cholecystitis died, and 1 perforation required surgery. On the other hand, a recent cost-effective analysis showed EUS-GBD had a higher total procedure cost per patient than PT-GBD. The cost of the EC-LAMS accounted for the major cost difference between the two procedures. EUS-GBD saved on the cost in management of AEs, reinterventions, and unplanned readmissions but these did not offset the cost of the stent (29).

### Obstruction of gastrointestinal tract

For patients with gastric outlet obstruction (GOO) or malignant stricture of duodenum who are not candidates of surgeries, endoscopic ultrasound (EUS)-guided gastrointestinal anastomosis with LAMS can be considered when gastrointestinal stents are unsuccessfully placed. This technique was first described in 2012 in a porcine model and was then reported promising results in humans (30, 31). When EC-LAMS was introduced, the delivery system was advanced directly into the adjacent gut lumen over the guidewire (32). EUS-guided gastrointestinal anastomosis with EC-LAMS was preferred for its shorter procedure time when compared with balloon-assisted approach. The technique success rate was reported of 80–94.5%, clinical successful rate was of 72.3–92.7% with AE rates of 6.5–14.3% (32, 33). Its success mainly depends on the distance between the two lumina that are going to be connected by the EC-LAMS and is influenced by the experience of endoscopist. Although this technique was thought to be useful in daily clinical practice, organizational challenges were considered to be the biggest obstacles that affect the diffusion of the procedure in about 55.2% of participants in a recent Italian survey (34).

### Gastric access temporary for endoscopy (GATE)

Another advantage of EC-LAMS is that it can quickly and accurately establish an access between adjacent gastrointestinal tracts. With its wide lumen, it can act as a working channel to allow an endoscope to pass through for further treatments on lesions in the gastrointestinal tract located in a long distance, thus significantly expanding the scope and breadth of endoscopic therapy (35). This technique is more focused on applying in patients who receive endoscopic retrograde cholangiopancreatography (ERCP) treatments with post-surgery anatomical changes like Roux-en-Y gastric bypass. Technical success rates can achieve 96% and persistent fistulas may occur in 11.7% patients, but endoscopic closure seems to be effective (36).

### Drainage of intra-abdominal fluids

As EC-LAMS is mastered by more endoscopists, its indications are also expanding. Drainage of many different types of intra-abdominal fluids can also be achieved by EC-LAMS, such as abdominal abscess (37–39). During these case series, EUS-guided transrectal drainages (EUS-TRD) of pelvic fluid collections with EC-LAMS were successfully performed in all cases and the stents were removed about 2 weeks after the placement without any adverse event or recurrence. Although some meta-analysis showed that EUS guided pelvic abscess drainage proves long-term clinical success with an acceptable rate of complications, the conclusion was drawn without regarding the difference between LAMS and plastic stents (40). Poincloux et al. (41) pointed out that, among the four patients who underwent LAMS for drainage of pelvic abscess, perforation and recurrence of abscess occurred in two patients, respectively, demonstrating LAMS did not achieve a perfect effect. Therefore, more clinical studies are needed to clarify the effectiveness and safety of EC-LAMS in the drainage of pelvic fluids.

## Complications of EC-LAMS

### Bleeding

Resent researches showed EC-LAMS was safe and had low risks in bleeding (42, 43), but there were still some case reports of delayed hemorrhage caused by LAMS (44). Delayed bleeding of LAMS placement when observed mostly due to underlying coagulopathy. One of the rare but life-threatening side effects of LAMS is delayed bleeding due to ruptured pseudoaneurysm (PA) (44). About 43.6% of patients had LAMS placed before PA diagnosis and bleeding from PA induced by erosion of LAMS may occur in the first 2 weeks (45). A possible mechanism for delayed bleeding in LAMS is its double-flange design. The two flanges make the gastric wall tightly close to the pseudocyst wall. After cystogastrostomy, the size of the pseudocyst is decreased because of the fluid drained from the pseudocyst into the gastric cavity. The double-flange design does not allow movement of walls or the stent. Lack of mobility may cause tension in the blood vessel wall and surrounding vessels, leading to PA formation and bleeding. About 4 weeks after LAMS implantation, the size of the cyst decreased significantly, and the possibility of delayed bleeding increased. Bang et al. (46) compared AXIOS and plastic stent for cystogastrostomy, and found that patients using plastic stent did not have delayed bleeding. They proposed that, unlike LAMS, with the collapse of WON, the plastic stent will enter the stomach freely. Brimhall et al. (47) and Lang et al. (17) both reported that patients with LAMS had a higher risk of pseudoaneurysm bleeding than patients with double-pigtail plastic stents in treating PFCs.

### Stent migration

LAMS was originally designed in a saddle shape to tightly connect the gut lumen with the cystic lesion together and minimize the risk of stent migration. However, the migration rate of LAMS had been reported by some studies in a range of 10–19% (48–50). Migration can occur immediately due to improper deployment of the LAMS, but may also occur weeks after stent placement, and also due to subsequent manipulation of the stent during the GATE procedure (50, 51). LAMS can migrate either into the cyst cavity, or back into the gastrointestinal lumen. The management of stent migration into the gastrointestinal lumen is mostly direct endoscopic extraction. Migration into the cyst cavity might lead to tract collapse and procedure failure, which should be managed by urgent endoscopic retrieval or surgery. In patients undergoing EUS-guided choledochoduodenostomy using an EC-LAMS, once the intra-channel release of the proximal flange from the duodenal bulb is not in a precise control, the pylorus could be completely covered by the proximal flange released transpylorically into the stomach, causing a rare complication of pyloric occlusion (52). The proximal flange was pushed in the right position by the gastroscope with a preloaded transparent cap.

### Buried stent

Buried stent refers to the condition that the stent ends pulled in and embedded into the stomach wall. This complication probably mainly occurs in LAMS, whether or not it is electrocautery enhanced, because the flanged edge of the stent is tightly contacted with the gastric and cyst wall. Several previous studies have reported no occurrence of this complication (43, 49, 53), but in other study, the rate of buried stent was reported of nearly 17% (46). The specific cause of this complication is not clear. Most of the buried stents were case reports, but in one recent review concerning complications of LAMS, occurrence rates of buried stent in PFC, bile duct and gallbladder were 0.07%, 0 and 0.59%, respectively (54).

## Application prospect in future

Currently, a porcine pilot study of gastric bypass bariatric surgery assisted by EC-LAMS has been successfully carried out (55). It is believed that this new technique can be applied into clinical practice in the near future, bringing good news to more obese patients.

## Author contributions

HY wrote the review. QL, S-hW, X-dG, and LZ search the related paper. BN gave the idea of this topic and revised the review. SH and BN revised the review. All authors contributed to the article and approved the submitted version.

## Funding

This work was supported by the Chongqing Natural Science Foundation project (CSTB2022NSCQ-MSX0130).

## Conflict of interest

The authors declare that the research was conducted in the absence of any commercial or financial relationships that could be construed as a potential conflict of interest.

## Publisher's note

All claims expressed in this article are solely those of the authors and do not necessarily represent those of their affiliated organizations, or those of the publisher, the editors and the reviewers. Any product that may be evaluated in this article, or claim that may be made by its manufacturer, is not guaranteed or endorsed by the publisher.
